# Effect of TGF-*β*1-Mediated Exercise Analgesia in Spared Nerve Injury Mice

**DOI:** 10.1155/2022/7382327

**Published:** 2022-10-19

**Authors:** Xinzheng Sun, Chenghao Wang, Junqi Wu, Xiaoke Chen, Hui He

**Affiliations:** ^1^School of Sports Science, Beijing Sport University, Beijing 100084, China; ^2^China Institute of Sports and Health, Beijing Sport University, Beijing 100084, China

## Abstract

Peripheral nerve injury leads to severe neuropathic pain. Previous studies have highlighted the beneficial effects of physical exercise on alleviating neuropathic pain. Exercise regulating transforming growth factor-*β*1 (TGF-*β*1) can improve several diseases and relieve neuropathic pain induced by peripheral nerve injury. Here, we investigated whether exercise could alleviate neuropathic pain by modulating TGF-*β*1 expression. We assessed mechanical and cold pain behavior and conducted molecular evaluation of the spinal cord. We found that spared nerve injury (SNI) led to mechanical and cold allodynia in the hind paw, elevated the expression of latency-associated peptide- (LAP-) TGF-*β*1, and activated astroglial in the spinal cord. Exercise decreases allodynia, astroglial activation, and LAP-TGF-*β*1 in SNI mice. Intrathecal injection of a TGF-type I receptor inhibitor attenuated exercise analgesia and enhanced astroglial activation. These findings demonstrate that exercise induces analgesia by promoting TGF-*β*1 activation and inhibiting astrogliosis. Our study reveals a new underlying mechanism for exercise-attenuated neuropathic pain in the maintenance stage of neuropathic pain after nerve injury.

## 1. Introduction

The incidence of neuropathic pain in the general population is as high as 3%–17% [1]. Neuropathic pain state is characterized by hyperalgesia, allodynia, and spontaneous pain, which greatly harm the well-being of the patient [2]. The pathogenesis of neuropathic pain is multifactorial, and the clinical therapeutic effect is not satisfactory. Drugs are the primary way of treating neuropathic pain, but the effect of drug treatment is limited, and there are many side effects; thus, it needs to be treated with other therapies. Numerous studies show that physical activity not only alleviates neuropathic pain but also improves functional outcomes [3].

Numerous studies have revealed that activation of microglia and astrocytes in the spinal plays a pivotal role in the development and maintenance of chronic pain [2, 4–6]. Reactive response astrocytes and microglia undergo morphological, molecular, and functional changes that are called astrogliosis and microgliosis, respectively [4, 5]. Neuropathic pain was attenuated by inhibiting the activation of astrocytes and microglia in the spinal cord dorsal horn of rodents [2, 6, 7]. Recent studies have shown that exercise can ameliorate astrogliosis and microgliosis to improve neuropathic pain [8, 9].

Transforming growth factor-*β*1 (TGF-*β*1), a multifunctional cytokine, regulates the transmission of nociceptive information [10]. TGF-*β*1 plays different roles in several types of pain. Some studies have shown that TGF-*β*1 acts on TGF-*β* receptor-I (TGF*β*RI-) mediated inflammatory and bone cancer pain [11, 12]. On the other hand, TGF-*β*1 can relieve neuropathic pain induced by peripheral nerve injury [13–16]. The mechanisms of TGF-*β*1 lessen neuropathic pain through the inhibition of activated astrocytes and microglia [13, 15].

Studies have shown that exercise can regulate TGF-*β*1 to improve several diseases [17, 18], but the role of TGF-*β*1 on exercise analgesia is unclear. This study investigated the effect of TGF-*β*1 in exercise analgesia by observing the effect of exercise on pain behavior in spared nerve injury (SNI) mice. We found that exercise can promote the activation of latent TGF-*β*1, inhibit the activation of astrocytes, and relieve mechanical and cold allodynia.

## 2. Materials and Methods

### 2.1. Animal and Experimental Design

Adult male C57BL/6J mice, 6–8 weeks and weighing 20–25 g, were provided by Beijing Keyu Animal Breeding Center (Beijing, China). The mice were placed in an environment at 22 ± 1°C with a 12-hour light-dark cycle and free access to food and water. The experimental protocol was approved by the Experimental Animal Welfare Ethics Committee of Beijing Sport University (Beijing, China) and the International Association for Pain Study under the guidance of the National Institutes of Health.

**Figure 1:**
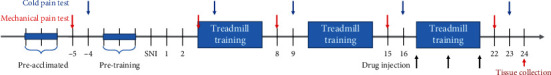
Schematic timeline for behavior detection and treadmill exercise. The red arrow represents the mechanical pain test. The blue arrow represents the cold pain test. The black arrow represents the drug injection time. The numbers represent the days of spared nerve injury surgery. Preacclimated was performed 30 min/day in pain test environment. Pretraining was performed 10 m/min/10 min/day. Treadmill training was performed 10 m/min/30 min/day/5 days/3 weeks.

Mice were preacclimated to the experimental environment (room and apparatus) and treadmill training before SNI surgery (see Figure 1). The exercise protocol was initiated three days after SNI surgery and continued for three weeks. When the pain tests were performed on the same day as the exercise, the pain tests were performed first. To clarify the effects of treadmill exercise on neuropathic pain, mice were divided into four groups and randomly assigned for (1) sham operation and sedentary animals (sham group), (2) spared nerve injury (SNI group), (3) spared nerve injury with exercise training (SNIE group), and (4) sham-operated animals with exercise training (shamE group). To investigate the effects of TGF-*β*1 on exercise analgesia, SNI with exercise training mice were divided into two groups and randomly assigned for (1) spared nerve injury with exercise training followed by intrathecal (i.t.) injection of saline (SNIE+saline) and (2) spared nerve injury with exercise training followed by i.t. injection of the TGF*β*RI inhibitor (SNIE+SB431542).

### 2.2. Spared Nerve Injury Model

Surgeries were performed as previously described [19]. Eight-week-old C57BL/6J mice were anesthetized by intraperitoneal injection of avertin (250 mg/kg; T48402, Sigma-Aldrich). After anesthesia, the right leg skin was cut open, and the muscles were bluntly separated to expose the tibial nerve, common peroneal nerve, and sural nerve. The common peroneal and tibial nerves were tightened with 5.0 silk, and the distal nerve stump was resected with 2-4 mm. Avoid contact or stretching of the intact sural nerve. The muscle and skin were closed in two layers. The sham mice underwent the same procedure as the experimental group, except the nerves, were left intact.

### 2.3. Exercise Protocol

The exercise protocol was performed according to previously described methods [20]. Three days before surgery, pretraining of all group animals were performed 10 m/min/10 min per day. Three days after surgery, exercise group animals were trained on a treadmill for 30 min per session at a speed of 10 m/min, five consecutive days for three weeks, with two resting days between each week. They were trained between 10 am and 12 pm per session. The sham operation and SNI groups were placed in the apparatus without exercise.

### 2.4. Mechanical Allodynia

For three consecutive days before the SNI surgery, the animals were placed in a plastic cage (6 × 8 × 12 cm) on a wire mesh platform to acclimate for at least 30 min per day. A series of von Frey filament series with logarithmic increasing stiffness was used in the test. The up-down method of von Frey monofilament testing was used in this study [21]. Briefly, von Frey filaments were applied to the ipsilateral side of the hind paw for a maximum duration of 4 s, and a withdrawal response was observed. Start with 0.16 g of stimulation. When a withdrawal response to a given filament was observed, a one-step weaker stimulation was applied. The procedure was repeated with sequentially thinner filaments until no paw withdrawal was observed. At this point, a one-step stronger stimulation was used again to confirm the paw withdrawal. The testing involved four more stimuli after the first change in response occurred, and the final score was converted to a 50% von Frey threshold according to Dixon and used for data analysis [22].

### 2.5. Cold Allodynia

Using a blunt needle connected to a syringe, carefully drop one drop of acetone solution (50 *μ*L) onto the lateral surface of the foot without touching the skin. The duration of lifting/licking was recorded. The interval of each test was at least 20 min, and the average value of the three tests was taken as the measurement result [23].

### 2.6. Intrathecal Injection

The syringe was inserted into the intervertebral space of a conscious mouse between the lumbar 5 (L5) and 6 (L6) regions of the spinal cord. A reflexive flick of the tail was considered an indicator of the accuracy of each injection [24]. The SB431542 (TGF*β*RI inhibitor, Selleckchem) diluted in saline was injected (100 *μ*mol/5 *μ*L) on days 17, 19, and 21 after the SNI surgery.

### 2.7. Immunofluorescence

Mice were deeply anesthetized by intraperitoneal injection of pentobarbital (100 mg/kg) and perfused transcardially with 0.9% saline followed by ice-cold 4% paraformaldehyde in 0.1 M phosphate buffer saline (PBS) (pH 7.4). The L4-L6 segments of the spinal cord were removed, postfixed at 4°C overnight, and placed in 20% sucrose solution for 24 h and 30% sucrose solution for 48 h at 4°C. The spinal cord was cut into 30 *μ*m thickness on a freezing microtome (Leica 9500, Germany). The sections were washed three times using PBS and blocked with Blocking Buffer for Immunol Staining (Beyotime, P0260) for 1 h at 37°C and then incubated overnight at 4°C with primary antibodies: Rabbit anti-GFAP polyclonal antibody (1 : 2000, ab7260, Abcam) and Rabbit anti-Iba1 (for Immunocytochemistry) (1 : 500, 019-19741, Wako). After incubation, the tissue sections were washed and then incubated with a secondary antibody (Alexa Fluor 488-conjugated donkey anti-rabbit IgG H&L, 1 : 1000, Invitrogen). After three rinses, all sections were cover-slipped using VectaShield. Images were obtained with a two-photon laser point scanning confocal microscopy system (Leica TCS SP8). The mean fluorescence intensity (MFI) in the entire spinal dorsal horn, including nine spinal slices from three mice in each group, was measured using Fiji software (ImageJ, NIH).

### 2.8. Western Blot Analysis

The L4-L6 segments were homogenized in tissue lysis buffer (Beyotime, P0013B) on ice. After centrifugation at 12,000 rpm for 20 min at 4°C, the supernatants were measured using a Pierce bicinchoninic acid (BCA) Protein Assay Kit (Thermo Scientific, Rockford, IL). The samples with sample loading buffer (Biotopped, Top2225) were heated for 10 min at 95°C, and equal amounts of protein (40 *μ*g) were separated by sodium dodecyl sulfate–polyacrylamide gel electrophoresis and then transferred onto PVDF membranes. The membranes were blocked with 5% skim milk in TBST (0.1% Tween 20 in 1× TBS) for 90 min at room temperature and then incubated overnight at 4°C with primary antibodies in blocking solution: mice-anti-TGF-*β*1 (1 : 1000, sc-130348, Santa Cruz, USA), rat-anti-TGF*β* receptor I (1 : 500, sc-101574, Santa Cruz, USA), rabbit-anti-GFAP (1 : 20000, ab7260, Abcam, USA), and rat-anti-Iba1 (1 : 500, sc-32725, Santa Cruz, USA). After three rinses in TBST, the membranes were incubated with appropriate horseradish peroxidase- (HRP-) linked secondary antibodies (anti-mouse IgG, HRP-linked antibody SA00001-1, 1 : 5000, Proteintech, China; anti-rat IgG, HRP-linked antibody ZB-2307, 1 : 5000, ZSGB, China; and anti-rabbit IgG, HRP-linked antibody ZB-2301, 1 : 5000, ZSGB, China) for 60 min at room temperature. The membranes were finally washed three times in TBST. Proteins were detected using the ECL Plus kit (ThermoFisher, USA) and ChemiDoc XRS+System (Bio-Rad). Blot was analyzed via image using Fiji software (ImageJ, NIH).

### 2.9. Statistical Analysis

Data are expressed as the means ± SEM. For the pain tests, differences between groups in mechanical allodynia and cold allodynia were determined using a two-way repeated-measures analysis of variance (ANOVA), followed by a post hoc least significant difference (LSD) multiple comparison test. For western blot and immunofluorescence, four group differences were determined using two-way ANOVA, followed by a post hoc LSD multiple comparison test. Student's *t*-tests were used for single comparisons between SNIE+saline and SNIE+SB431542. Differences were considered significant if *P* < 0.05.

## 3. Results

### 3.1. Treadmill Exercise Improves Neuropathic Pain Behavior

**Figure 2:**
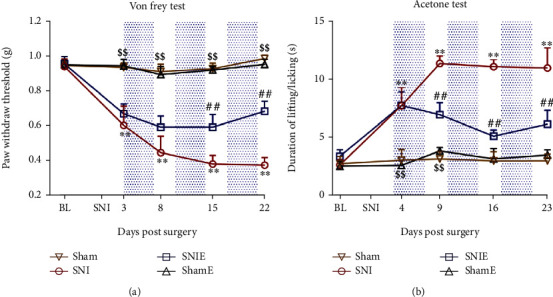
Treadmill exercises improve neuropathic pain behavior. (a) Mechanical allodynia test (*n* = 8). (b) Cold allodynia test (*n* = 8). Values represent the mean ± SEM; ^∗∗^*P* < 0.01 versus the sham group, ^##^*P* < 0.01 versus the SNI group, and ^$$^*P* < 0.01 versus the SNIE group.

To investigate the effect of treadmill exercise on neuropathic pain caused by SNI, the ipsilateral paw mechanical pain thresholds were measured on day 5 before and on days 3, 8, 15, and 22 after SNI surgery (Figure 2(a)). Compared with the sham group, the mice on day 3 after the SNI surgery displayed a lower paw withdrawal threshold (PWT) lasting from days 3 to 22 at least (*P* < 0.01). On days 15 and 22, the PWT in the SNIE group, which underwent treadmill training, was higher than that in the SNI group (*P* < 0.01). The lifting/licking responses to a cold stimulus were measured on day 4 before and on days 4, 9, 16, and 23 after the SNI surgery (Figure 2(b)). Compared with the sham group, the mice on day 4 after SNI surgery displayed a higher lifting/licking time lasting on day 23 (*P* < 0.01). At day 9, the duration of lifting/licking in the SNIE group was lower than that in the SNI group (*P* < 0.01). This decrease was sustained throughout the duration of the study. These results suggest that exercise can decrease SNI-induced mechanical and cold allodynia.

### 3.2. Exercise Promotes TGF-*β*1 Activation

**Figure 3:**
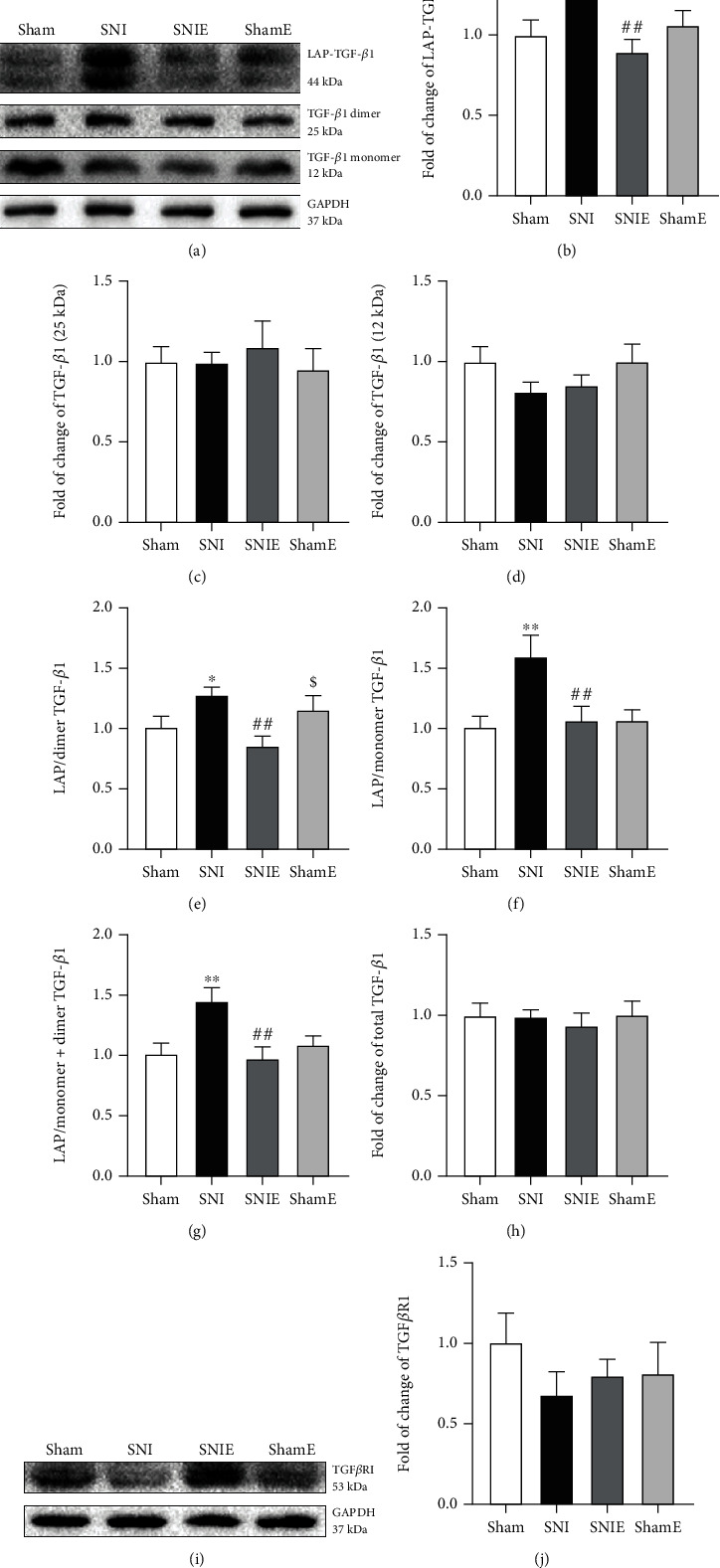
Exercise activates latent TGF-*β*1 in the spinal cord tissue of four groups of mice 24 days after SNI surgery. (a) Representative images of western blot for TGF-*β*1 monomer, TGF-*β*1 dimer, and LAP-TGF-*β*1. Western blotting analysis for the expression changes of (b) LAP-TGF-*β*1, (c) dimer TGF-*β*1 (25 kDa), and (d) monomer TGF-*β*1 (12 kDa) (*n* = 6). The ratio of (e) LAP-TGF-*β*1/dimer TGF-*β*1, (f) LAP-TGF-*β*1/monomer TGF-*β*1, and (g) LAP-TGF-*β*1/monomer+dimer TGF-*β*1 (*n* = 6). (h) Total TGF-*β*1: monomer+dimer+LAP-TGF-*β*1/GAPDH (*n* = 6). (i) Representative images of western blot for TGF*β*RI (*n* = 6). (j) Western blotting analysis for the expression changes of TGF*β*RI (*n* = 6). Values represent the mean ± SEM. ^∗^*P* < 0.05, ^∗∗^*P* < 0.01 versus the sham group; ^#^*P* < 0.05, ^##^*P* < 0.01 versus the SNI group; and ^$^*P* < 0.05 versus the SNIE group.

Latent TGF-*β*1 (LAP-TGF-*β*1) has a latency-associated peptide (LAP) structure that prevents mature TGF-*β*1 from binding to its receptor, which is inactive [22]. Mature TGF-*β*1 needs to be released from the LAP to be active [22]. Mature TGF-*β*1 is a homologous dimer formed by disulfide bonds. Part of the disulfide bonds of dimer TGF-*β*1 was broken in the process of the western blot experiment, and two structural forms of monomer (12 kDa) and dimer (25 kDa) were observed (Figure 3(a)). Western blot analyses showed that LAP-TGF-*β*1 protein levels in the SNI group were obviously increased compared with the sham group (*P* < 0.01). LAP-TGF-*β*1 expression was significantly reduced in the SNIE group compared with the SNI group (*P* < 0.01, Figure 3(b)). Monomer TGF-*β*1 and dimer TGF-*β*1 expressions were not significantly different between the sham, shamE, SNI, and SNIE groups (Figures 3(c) and 3(d)). To further explore the effect of exercise on TGF-*β*1 activation, the ratio of LAP/monomer TGF-*β*1 (*P* < 0.01), LAP/dimer TGF-*β*1 (*P* < 0.01), and LAP/monomer+dimer TGF-*β*1 (*P* < 0.01) in the SNI group represented higher values when compared to the sham group, and the ratio of LAP/monomer TGF-*β*1, LAP/dimer TGF-*β*1, and LAP/monomer+dimer TGF-*β*1 in the SNIE group was significantly lower than that in the SNI group (*P* < 0.01, Figures 3(e)–3(g)). There was no significant difference in total TGF-*β*1 and TGF*β*RI between each group (Figures 3(h)–3(j)). These results reveal that SNI increases LAP-TGF-*β*1 levels and exercise decreases LAP-TGF-*β*1 expression (TGF-*β*1 activation) in the spinal cord of SNI mice.

### 3.3. Exercise Inhibits Astrocyte Activation after SNI

**Figure 4:**
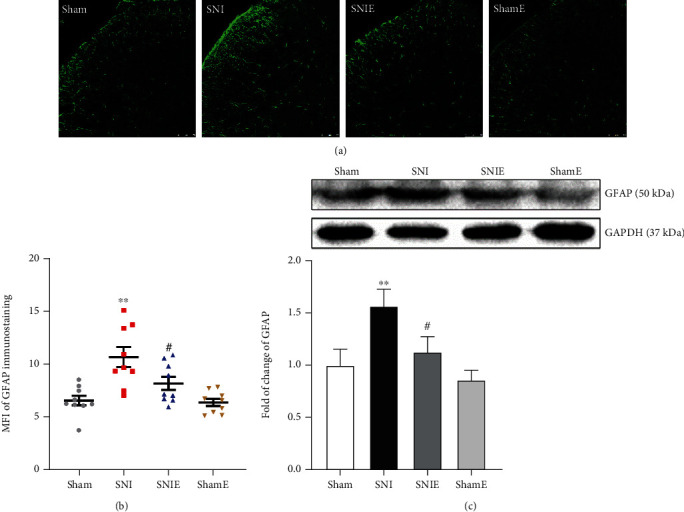
Astrocytes in the spinal cord are activated in mice 24 days after SNI. (a) Representative images of MFI for GFAP in the spinal dorsal horn. (b) Quantification of GFAP in the spinal dorsal horn (scar bar = 75 *μ*m, *n* = 9  spinal slices from three mice for each group). (c) Western blotting analysis for the expression changes of GFAP (*n* = 6). Values represent the mean ± SEM.  ^∗∗^*P* < 0.01 versus the sham group; ^#^*P* < 0.05 versus the SNI group.

**Figure 5:**
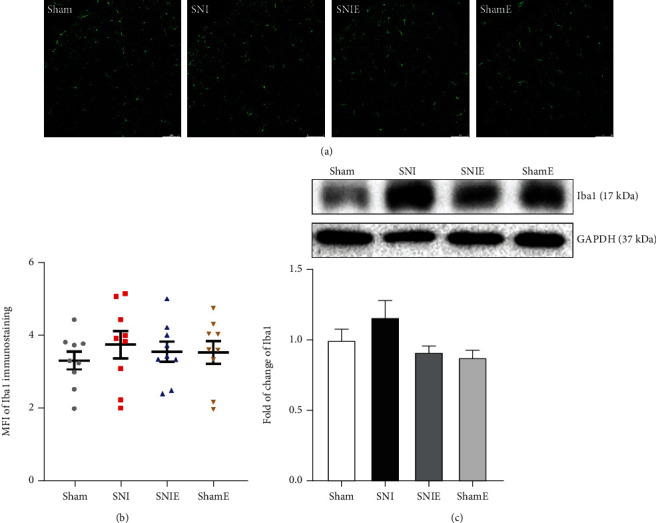
Microglia in the spinal cord are not activated in mice 24 days after SNI. (a) Representative images of MFI for Iba1 in the spinal dorsal horn. (b) Quantification of Iba1 in the spinal dorsal horn. Values represent the mean ± SEM (scale bar = 75 *μ*m, *n* = 9 spinal slices from three mice for each group). (c) Western blotting analysis of the expression changes of Iba1 (*n* = 6). Values represent the mean ± SEM.

Astrocyte and microglia activation in the spinal dorsal horn plays a fundamental role in the development and maintenance of neuropathic pain after nerve injury, while its activation characteristics are different in various types of pain [5, 23]. The expression of glial fibrillary acidic protein (GFAP) (an astroglial marker) and ionized calcium-binding adaptor molecule 1 (Iba1) (a microglia marker) was detected by immunofluorescence and western blotting. Immunofluorescence and western blot results showed that the expression of GFAP in the SNI group was significantly higher than that in the sham group (*P* < 0.01), and the expression of GFAP in the SNIE group was significantly lower than that in the SNI group (*P* < 0.05, Figures 4(b) and 4(c)). The expression of Iba1 in the spinal cord horn was not significantly different between the groups (Figures 5(b) and 5(c)). We can highlight that three weeks of exercise inhibits astrogliosis in the spinal cord of SNI mice.

### 3.4. TGF*β*RI Inhibitor Reverses Exercise Analgesia and Its Effect on Astrocyte Reactivity

**Figure 6:**
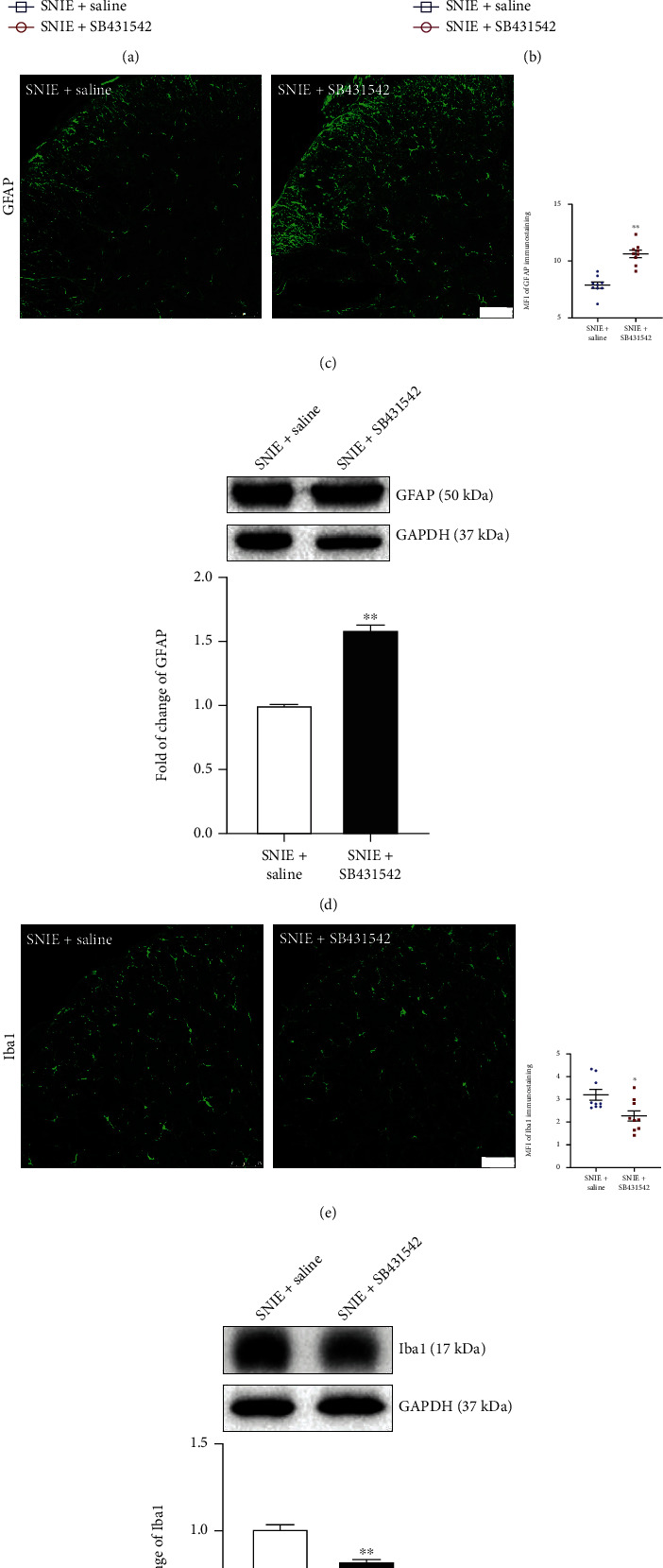
TGF*β*RI inhibitor reverses exercise analgesia. (a, b) Mechanical and cold allodynia tests (*n* = 8). The blue shade represents the period of the exercise intervention, and the gray arrow represents the time of injection. ^&^*P* < 0.05, ^&&^*P* < 0.01 versus the SNIE+saline group. (c) Quantification of GFAP in the spinal dorsal horn. Values represent the mean ± SEM (scar bar = 75 *μ*m, *n* = 9 spinal slices from three mice for each group). (d) Western blotting analysis for the expression changes of GFAP (*n* = 8).(e) Quantification of Iba1 in the spinal dorsal horn. Values represent the mean ± SEM(scar bar = 75 *μ*m, *n* = 9 spinal slices from three mice for each group). (f) Western blotting analysis for the expression changes of Iba1 (*n* = 8). Values represent themean ± SEM. ^∗^*P* < 0.05,  ^∗∗^*P* < 0.01versus the SNIE+saline group.

TGF-*β*1 can regulate pain signal transduction by acting on TGF*β*RI [9, 11, 12]. To further determine whether TGF-*β*1 is involved in exercise analgesia, we used TGF*β*RI inhibitor to block TGF-*β*1 signaling transduction in SNIE group mice to observe pain behavior and glial cell activation. Exercise training followed by intrathecal injection of drugs was performed on days 17, 19, and 21 after SNI surgery. After drug injection of SNI surgery with exercise-training mice, TGF*β*RI inhibitor treatment of the SNIE+SB431542 group aggravated mechanical allodynia (*P* < 0.01, Figure 6(a)) and cold allodynia (*P* < 0.05, Figure 6(b)) compared with 0.9% saline treatment of the SNIE+saline group. Immunofluorescence results showed that the expression of GFAP in the SNIE+SB431542 group was significantly higher than that in the SNIE+saline group (*P* < 0.01) (Figure 6(c)), and Iba1 was significantly different between the SNIE+SB431542 and SNIE+saline group (*P* < 0.05) (Figure 6(e)). Western blot results showed that the protein level of GFAP in the SNIE+SB431542 group was significantly higher than that in the SNIE+saline group (*P* < 0.01, Figure 6(d)), but the Iba1 protein level in the SNIE+SB431542 group was remarkably decreased compared with that in the SNIE+saline group (*P* < 0.01, Figure 6(f)).

## 4. Discussion

Here, we demonstrated that exercise alleviated mechanical and cold allodynia in a mouse model of SNI. Moreover, exercise normalized LAP-TGF-*β*1 upregulation in the spinal dorsal horn and reversed astrocyte hyperactivity after nerve injury. TGF*β*RI inhibitor reversed the effect of exercise analgesia and promoted astrocyte activation but inhibited microglia expression.

Mechanical and cold allodynia occurred on the 3rd and 4th days after SNI surgery, respectively, and the allodynia was maintained until the end of the experiment. Previous studies have shown that the SNI model induced mechanical and cold allodynia for 12 months [25], which is suitable for the study of the effect of long-term regular exercise on pain. Studies have shown that high-intensity exercise has a better analgesic effect than low-intensity exercise [26], but excessive high-intensity exercise may aggravate pain in mice [27]. Running at 10 m/min is equivalent to 75% lactate threshold intensity in mice [20] and could effectively relieve mechanical allodynia in mice with peripheral nerve injury. In addition to exercise intensity, exercise frequency is an important factor affecting the effect of exercise intervention on pain. Sumizono et al. [28] found that exercise intervention five times a week had a better effect on improving mechanical pain hypersensitivity than three times a week, indicating that higher frequency exercise had a significant effect on enhanced neuropathic pain caused by peripheral nerve injury in mice. Duration of exercise intervention is positively correlated with analgesic effect, and only 2-day centrifugal exercise can significantly improve cold pain hypersensitivity in mice with sciatic nerve injury [29]. In the present study, running on a treadmill at 10 m/min, five days/week, was used to effectively relieve cold allodynia after one week of exercise and mechanical allodynia after two weeks of exercise, which was consistent with the results of previous studies [20, 29].

Neuropathic pain is often accompanied by the activation of astrocytes and microglia [6], but the timing of activation is different. A study showed that astrocytes and microglia were activated significantly two weeks after peripheral nerve injury [9]. This study showed that spinal cord astrocytes were activated three weeks after SNI surgery but not microglia. Astrogliosis is evident one week after nerve injury in mice and lasts for many months [30]. In comparison, microgliosis is a transient and self-limited event. Spinal microglia begin to proliferate within 2 to 3 days and reach maximal levels in 4 to 7 days after peripheral nerve injury. However, the activated microglia return to normal levels within a few weeks [31, 32]. Previous studies showed that two weeks of treadmill exercise and five weeks of swimming exercise inhibited the activation of astrocytes in the spinal cord caused by peripheral nerve injury [8, 9]. This study also showed that three weeks of treadmill exercise also inhibited the activation of astrocytes in the spinal cord. This suggests that inhibition of astrocyte activation by exercise is an important mechanism for exercise analgesia.

TGF-*β*1 is initially produced as a precursor called pre-pro-TGF-*β*1. After multiple processes, latent TGF-*β*1 was formed. Latent TGF-*β*1 is inactive because latency-associated protein (LAP) prevents the binding of mature TGF-*β*1 to its receptor [33, 34]. TGF-*β*1 bioactivity requires the release of mature TGF-*β*1 from the LAP structure, a process referred to as TGF-*β*1 activation [33, 35]. In the western blot experiment, part of the dimer TGF-*β*1 disulfide bond was broken and was divided into two bands of monomer TGF-*β*1 (12 kDa) and dimer TGF-*β*1 (25 kDa) in gel electrophoresis. Our results showed that no statistical difference of mature TGF-*β*1 in the mouse spinal cord among all groups. However, the expression of LAP-TGF-*β*1 in the spinal cord of SNI mice was significantly increased, while exercise significantly inhibited the expression of LAP-TGF-*β*1. A recent study showed that increased LAP-TGF-*β*1 expression indicates decreased TGF-*β*1 activation [36], so exercise may reduce LAP-TGF-*β*1 levels to promote TGF-*β*1 activation. The ratio of LAP-TGF-*β*1/mature TGF-*β*1 showed that the value was significantly increased in the spinal cord of SNI mice and decreased by exercise. It showed that exercise could promote the activation of TGF-*β*1. Interestingly, although LAP-TGF-*β*1 does not directly interact with TGF-*β* receptors, it has a high affinity with neuropilin-1 [35], which may affect pain signal transduction by regulating other molecules.

TGF*β*RI inhibitor can block the analgesic effect of TGF-*β*1, which indicates that TGF-*β*1 acts on TGF*β*RI to exert an analgesic effect [13]. This study showed that exercise promoted the activation of TGF-*β*1 and inhibit the activation of astrocytes. Moreover, this study revealed that TGF*β*RI inhibitor promotes astroglial activation. And both TGF-*β*1 and TGF*β*RI are expressed in astrocytes [37–39]. This suggests that exercise-induced activation of TGF-*β*1 may alleviate neuropathic pain by inhibiting astrogliosis.

Many studies have shown that microglia activation is one of the important causes of neuropathic pain [4]. Western blot results showed that injection of the TGF*β*RI inhibitor inhibited the expression of microglia. However, the results of this study showed that the TGF*β*RI inhibitor can aggravate the mechanical and cold pain of SNIE mice. Butovsky et al. [40] found that TGF-*β*1 mediates the growth and differentiation of microglia, and the amount of microglia in the central nervous system of mice was significantly reduced after the TGF-*β*1 gene was knocked out. Together, these findings suggest that TGF*β*RI may inhibit the growth and differentiation of microglia.

## 5. Conclusions

Our study provides evidence that exercise promotes the activation of TGF-*β*1 and inhibits the activation of astrocytes, thereby alleviating mechanical and cold allodynia in SNI mice.

## Data Availability

All datasets generated for this study are available on request to the corresponding author.
